# Preliminary clinical outcomes of irreversible electroporation in the management of prostatic hyperplasia

**DOI:** 10.1097/MD.0000000000046166

**Published:** 2025-11-28

**Authors:** Zhiyu Yu, Quan Wen, Yunpeng Guo, Long Chen, Yuyang Wang, Gai Hang, Bo Chen

**Affiliations:** a Department of Urology, The First Hospital of China Medical University, Shenyang, China; b Department of Urology, Tongliao People’s Hospital, TongLiao, China; c Graduate School of Inner Mongolia Medical University, Hohhot, China.

**Keywords:** irreversible electroporation, prostate-specific antigen, prostatic hyperplasia

## Abstract

This study evaluates the efficacy and safety of IRE (irreversible electroporation) ablation in the management of BPH (benign prostatic hyperplasia). Clinical data from 9 patients with prostate hyperplasia who underwent IRE treatment between September 2022 and August 2024 were retrospectively analyzed. Outcomes were assessed to determine the effectiveness and safety of IRE in ablating hyperplastic prostate tissue. IRE ablation was successfully performed in all patients. Postoperative assessments showed no significant reduction in prostate volume compared to preoperative measurements, This might be related to the mechanism of action of IRE. However, maximum urine flow rate significantly increased, and PVR (residual urine volume) significantly decreased. Additionally, the IPSS (International Prostate Symptom Score) showed a statistically significant improvement (*P* < .05) compared to preoperative values. Sexual function remained largely unaffected. Adverse reactions were reported in 2 cases (22%). IRE has demonstrated better efficacy and an acceptable safety profile, accompanied by a low incidence of postoperative complications in the treatment of BPH. This technique provides a novel alternative for clinical management. However, due to the limited sample size in this study, further validation through long-term follow-up studies and expanded cohorts is necessary to confirm its sustained effectiveness.

## 1. Introduction

BPH (benign prostatic hyperplasia) is a prevalent urological condition in middle-aged and elderly males.^[1]^ It is characterized by progressive enlargement of the prostate gland, leading to LUTS (lower urinary tract symptoms) such as urinary frequency, urgency, and retention.^[2]^ These symptoms significantly impair quality of life and may also compromise renal function if left untreated. Pharmacological therapies are commonly used in early stages but are not definitive for long-term management. Surgical intervention remains the ultimate treatment to alleviate refractory LUTS, prevent complications, and restore urinary function. Currently, TURP (transurethral resection of the prostate) and TULIP (transurethral laser procedures) are standard surgical options for BPH management. While effective, these procedures have inherent limitations, such as extended operative times, substantial intraoperative blood loss, and risks of postoperative complications, including urinary incontinence, erectile dysfunction, and prolonged recovery periods.^[3]^ The drawbacks of conventional approaches have driven the exploration of novel, minimally invasive techniques to improve outcomes and reduce morbidity. Irreversible electroporation (IRE), also known as the nanoknife technique, is an emerging minimally invasive modality originally developed for the focal treatment of malignant tumors, including liver and prostate cancers. This technology uses electrical pulses to disrupt the lipid bilayer of cell membranes, inducing irreversible nanoscale (80–490 nm) electroporation. The process triggers controlled cellular apoptosis within 1 to 7 days, resulting in precise tissue ablation without significant thermal injury. Despite its successful application in oncology, the use of IRE in the management of BPH remains underexplored. This retrospective study evaluates the clinical outcomes of 9 patients with BPH who underwent IRE ablation, offering a preliminary assessment of its efficacy and safety as a potential alternative to conventional surgical techniques.

## 2. Materials and methods

### 2.1. Clinical data

A retrospective analysis was conducted on a cohort of 9 patients who presented with prostate hyperplasia between September 2022 and August 2024, and subsequently underwent IRE ablation techniques. All patients underwent preoperative examination to confirm the diagnosis of prostatic hyperplasia, independently opted for IRE surgery, and provided informed consent for the procedure. The surgical procedures were exclusively conducted by urologists possessing advanced surgical expertise. In this study, a comprehensive review of clinical data was undertaken to assess the postoperative outcomes of patients. This research was approved by the Ethics Committee of Tongliao People’s Hospital.

Inclusion criteria were as follows: signed informed consent; diagnosis of BPH confirmed by imaging examination; PSA (prostate-specific antigen) level < 4 ng/mL; and patients with IRE surgical indications.

Exclusion criteria: Individuals with a history of mental illness or those unable to cooperate with the procedure; Patients diagnosed with prostate cancer; Presence of lower urinary tract malformations or injuries; Severe organ failure rendering them unsuitable for surgery; Patients who have been on long-term anticoagulant medication and discontinued its use for less than 1 week.

### 2.2. Surgical methods

#### 2.2.1. Stitch principles

**Figure 1.**
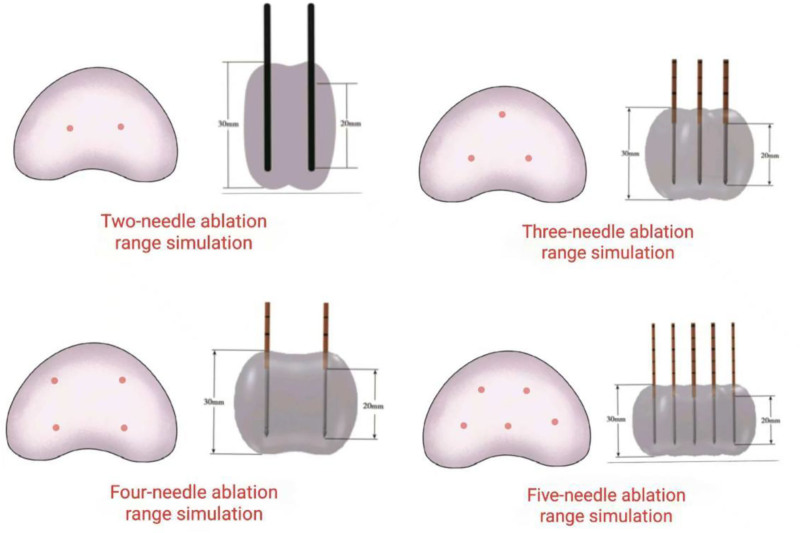
Schematic diagram of ablation.

Longitudinal axis: the needle body penetrates the tissue, with both the front and back ends exposed more than 0.5 cm each, in order to avoid protrusion beyond the prostate capsule. Cross section: center surround method: the central needle is positioned at the center of the lesion, while the surrounding needle (surrounding electrode) is placed 0.5 to 2 cm away from the central needle based on gland size. Surrounding method: In cases where the gland is small and it is not possible to maintain a distance >0.5 cm between 2 electrodes, a wrapping technique is employed by encircling the lesion with at least 0.5 cm margin within the gland’s edge. Please refer to Figure 1 for further details.

#### 2.2.2. IRE ablation methods

**Figure 2.**
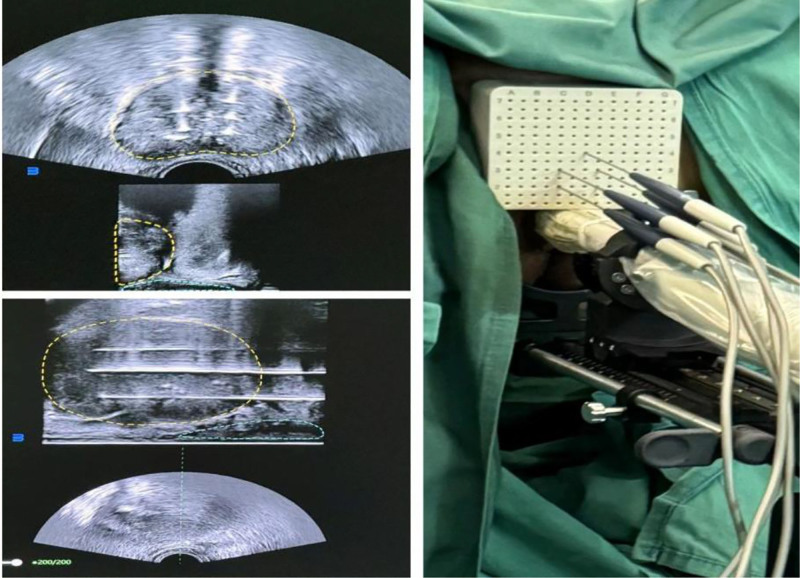
Intraoperative ultrasonic positioning operation.

IRE ablation was performed under ultrasound (USG) guidance, targeting the hyperplastic prostate tissue. The system’s software allowed adjustment of parameters such as electric field intensity, pulse number, pulse width, electrode number, and spacing to optimize electrode exposure.^[4]^ A control platform generated a plane graph perpendicular to the ablation electrode, delineating the precise ablation range. The operating platform was configured with parameters including an electric field intensity of 1500 to 3000 V/cm, 100 to 150 pulses, a pulse width of 70 to 90 μs, electrode spacing of 1.5 to 2 cm, and an electrode exposure length of 1.5 to 2 cm.^[5–8]^ The number of probes, needle insertion methods, and intraoperative parameters were determined based on the preoperative treatment plan. During the procedure, 2 to 3 electrodes were inserted into the targeted prostate area with an effective exposure length of 1 to 2 cm and interelectrode spacing of 1 to 2 cm. USG-guided needle placement was performed to ensure precision, and ablation was initiated using an ECG-synchronized mode to deliver pulses within the ventricular refractory period, minimizing the risk of arrhythmias. Treatment plan software simulated the ablation range and monitored cardiac electrical activity. After delivering a test pulse, the remaining pulses were output continuously to complete the cycle. For large prostates, multiple zonal ablations were performed, each lasting approximately 10 minutes. Upon completion, the electrodes were carefully withdrawn, and USG examination confirmed successful ablation through visible changes in the treated area. Please refer to Figure 2 for further details.

### 2.3. Observation indicators

The patient’s age, operation time, intraoperative blood loss, postoperative hospital stay, postoperative catheter removal time, preoperative and postoperative prostate volume, Qmax (maximum urinary flow rate), PVR (residual urine), IPSS (international Symptom score of prostatic hyperplasia), PSA and postoperative adverse reactions were observed. IIEF-5 (International Erectile Function Index 5) and RE (retrograde ejaculation) were used to evaluate the sexual function of the patients before and 6 months after surgery. Short-term effectiveness assessment included measurements of PSA levels and multiparameter MRI changes. Multiparameter MRI was reviewed 6 months after surgery. The MRI sequences of all patients included T2-weighted imaging, dynamic enhanced imaging, diffusion-weighted imaging and PET-like imaging. The images were reviewed separately by an experienced chief physician who had been engaged in imaging diagnosis for many years.

### 2.4. Statistical methods

In this study, the SPSS 20.0 statistical software, Chicago was used for data processing and analysis, and the significance level was set at α = 0.05. For continuous variables, data conforming to the normal distribution were expressed as X ± S. For inter-group comparisons, the independent samples *t* test was used for data conforming to the normal distribution, the rank sum test was used for skewed distributions, and the Chi-square test (χ^2^) or Fisher exact probability method was selected for categorical data.

## 3. Outcomes

Patient parameters, including age, operation time, intraoperative blood loss, postoperative hospital stay, catheter removal time, prostate volume (pre- and postoperative), Qmax, PVR, IPSS, PSA, and postoperative adverse reactions, were recorded. Sexual function was evaluated using the IIEF-5 and the presence of RE both preoperatively and 6 months postoperatively. Short-term outcomes were evaluated based on changes in PSA levels and findings from multiparametric MRI.

### 3.1. Patient’s age, operation time, postoperative hospitalization time, and postoperative catheter extraction time

The mean age of the 9 patients was (59 ± 2.29) years, with an average operation time of (76.1 ± 4.17) minutes, needle placement time of (22.11 ± 3.06) minutes, and ablation time of (22.22 ± 2.64) minutes. Intraoperative blood loss averaged at (1.44 ± 0.53) mL, postoperative hospital stay lasted for an average of (4.33 ± 1.32) days, and postoperative catheter removal occurred after an average duration of (17.89 ± 5.75) days as shown in Table 1.

**Table 1:** The patient’s age, operation time, postoperative hospitalization time, postoperative catheter removal time.

Item	Result (X ± S)
Age, yr	59 ± 2.29
Operation time, min	76.1 ± 4.17
Needle time, min	22.11 ± 3.06
Ablation time, min	22.22 ± 2.64
Intraoperative blood loss, mL	1.44 ± 0.53
Postoperative hospital stay, d	4.33 ± 1.32
Catheter removal time after operation, d	17.89 ± 5.75

### 3.2. Comparison of prostate volume, Qmax, PVR, IPSS, and IIEF-5 before and after surgery

postoperatively, there was no significant reduction in prostate volume; however, a statistically significant increase was observed in the Qmax, along with a notable decrease in PVR and a significant improvement in the IPSS (*P* < .05), no significant changes were noted in the IIEF-5. Please refer to Table 2 for detailed information.

**Table 2:** Comparison of prostate volume, Qmax, PVR, IPSS and IIEF-5 before and after surgery.

Item	Before operation (X ± S)	6 mo after surgery (X ± S)
Prostatic volume (cm^3^)	64.96 ± 17.28	57.94 ± 17.54
Qmax (mL/s)	5.37 ± 1.53	19.93 ± 3.16
PVR (mL)	130.67 ± 66.74	3.67 ± 2.55
IPSS	26 ± 2.65	5.44 ± 1.13
IIEF-5	20.44 ± 2.3	20.56 ± 2.46

IIEF-5 = International Index of Erectile Function-5, IPSS = International Prostate Symptom Score, PVR = residual urine volume, Qmax = maximum urinary flow rate.

### 3.3. Specific indicators: PSA and RE

The immediate postoperative PSA examination of the patient revealed a remarkable surge in PSA levels, surpassing 100 + (ng/mL). Subsequent follow-up examination conducted 6 months after surgery indicated a return to preoperative PSA levels. Notably, no instances of RE were observed among all patients. Please refer to Table 3 for detailed information.

**Table 3:** Comparison of PSA and RE before and after surgery.

Item	Before operation (X ± S)	Postoperation (X ± S)	6 mo after surgery (X ± S)
PSA	1.06 ± 0.68	179.22 ± 27.88	0.98 ± 0.55
RE	0	0	0

PSA = prostate-specific antigen, RE = retrograde ejaculation.

### 3.4. Preoperative and postoperative imaging findings

**Figure 3.**
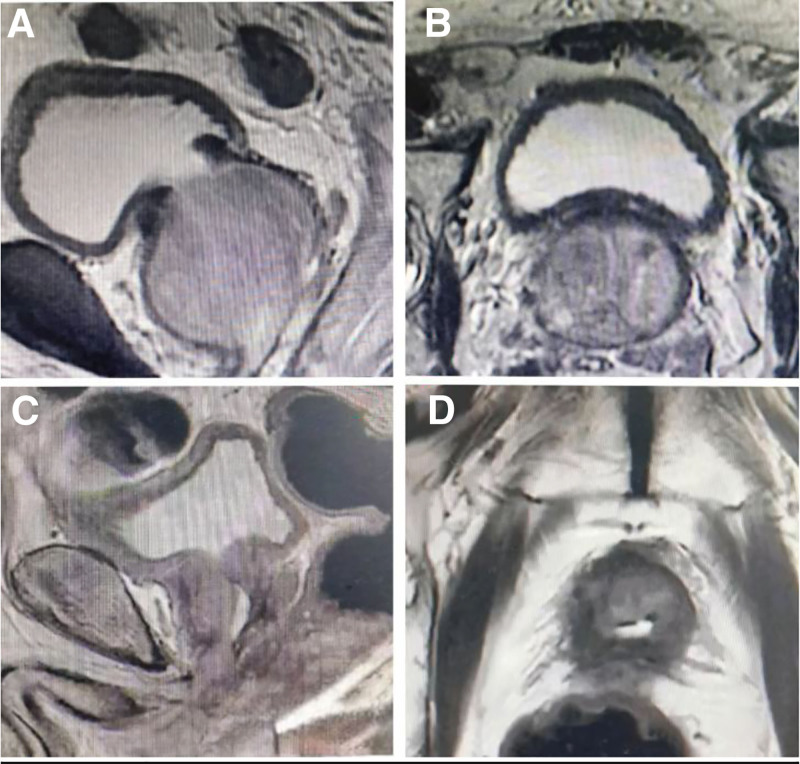
(A) Preoperative sagittal plane. (B) Preoperative cross section. (C) Postoperative sagittal plane. (D) Postoperative cross section.

All patients were diagnosed with prostatic hyperplasia through preoperative imaging techniques. The MRI results revealed a clear demarcation between the anterior central gland and bilateral peripheral band, an uneven signal in the central gland, and no abnormalities in the bilateral seminal vesicles. Postoperative MRI demonstrated irregularities in the prostatic region following prostate surgery. Please refer to Figure 3 for further details.

### 3.5. Postoperative adverse reaction

Urinary incontinence, scrotal edema, and difficulty defecating were not observed after surgery. Two patients had urinary retention, which recovered before discharge after symptomatic treatment. One patient experienced transient erectile dysfunction and recovered 3 months after surgery. There were no postoperative complications such as hematuria, urinary tract infection and rectourethral fistula reported in the literature.

## 4. Discussion

BPH is a prevalent urological condition in clinical practice. While it poses minimal threat to the patient’s life due to its slow progression, it can lead to significant urinary tract obstruction, resulting in renal function impairment. Prolonged obstruction also increases the risk of secondary infections, thereby impacting patients’ immune function.^[9]^ Furthermore, as living standards continue to improve, the age of onset of BPH progressively decreases, thereby increasing patients’ expectations regarding postsurgical preservation of sexual function. TURP currently represents the clinical “gold standard” for BPH treatment. Despite its widespread use in clinical practice, some patients still experience postoperative complications such as urinary incontinence and RE.^[10]^

The IRE technique is a novel minimally invasive ablation method that utilizes ultra-short pulses under a high-voltage electric field to selectively disrupt the phospholipid bilayer of cell membranes. This process induces transmembrane potential, resulting in the formation of nanoscale hydrophilic pores on the cell membrane, ultimately causing its structural collapse. The permanent porosity disrupts cellular homeostasis, leading to uncontrolled leakage of intracellular components or delayed membrane closure, thereby causing irreversible cellular injury. Unlike thermal or radiation-based ablation methods that induce necrosis, IRE triggers controlled apoptosis. This mechanism preserves surrounding normal tissues, including peripheral nerves and blood vessels, minimizing collateral damage while effectively ablating targeted lesions.^[11]^

IRE offers the potential to preserve connective tissue and adjacent critical structures, including neurovascular bundles, blood vessels, and biliary ducts, making it a promising approach for the ablative treatment of prostate hyperplasia.^[12–14]^ Unlike thermal ablation techniques, IRE effectively prevents incomplete ablation of perivascular or urethral tissues caused by heat sink effects and minimizes damage to temperature-sensitive structures such as peripheral nerves and blood vessels. Additionally, the short duration of IRE pulses reduces overall procedure time. The absence of surgical incisions in IRE minimizes intraoperative bleeding, accelerates patient recovery, and reduces postoperative complications, thereby enhancing the safety and efficacy of the treatment. In this study, the average operation time was 76.1 ± 4.17 minutes, which included a needle placement time of 22.11 ± 3.06 minutes and an ablation time of 22.22 ± 2.64 minutes. The average intraoperative blood loss was 1.44 ± 0.53 mL, and the postoperative hospital stay was 4.33 ± 1.32 days. Compared to conventional TURP and TULIP procedures, IRE reduced operative time by nearly 50%, with a significant portion of the time dedicated to preoperative preparation. This technique also minimized intraoperative blood loss, reducing the risk of surgical complications, enhancing postoperative outcomes, and allowing for expedited hospital discharge. IRE is particularly suitable for patients with coagulopathies or those with contraindications to TURP or TULIP procedures. Postoperative evaluations revealed no significant change in prostate volume compared to preoperative measurements. However, significant improvements were observed in functional outcomes, including an increase in Qmax, a decrease in PVR, and improved IPSS.

The findings of this study demonstrated that most patients experienced no significant changes in erectile function following IRE treatment Only 1 patient reported transient erectile dysfunction, which fully resolved within 3 months. No cases of RE were observed during the follow-up period. The impact of IRE on sexual function was first investigated by Onik et al^[15]^ in a canine model. Despite the urethra being within the ablation zone, its structural integrity remained unaffected post-ablation. Kim et al^[16]^ further evaluated the effects of IRE on erectile function, adverse events, and surrounding tissues in a bilateral prostate model in dogs. Pathological analysis revealed no evidence of erectile dysfunction in any of the 12 animals, with no damage observed in major arteries, veins, or adjacent tissues. A 5-year follow-up study by Scheltema et al^[17]^ reported that 99% of patients treated with IRE retained good urinary control, while erectile function recovered in up to 95% of patients within a maximum of 23 days post-procedure. Similarly, a recent study by Yan et al^[18]^ found that while short-term adverse effects on sexual function may occur following IRE, the technique effectively preserves urinary function and quality of life while reducing perioperative complications. Postoperative PSA evaluation revealed a marked increase in PSA levels (preoperative values of 1.06 ± 0.68 ng/mL to over 100 ng/mL). This significant surge is consistent with the mechanism of IRE, which induces cell membrane poration and subsequent cell disintegration, leading to the release of intracellular contents, including PSA. Notably, this represents a novel observation, as such a pronounced postoperative elevation in PSA levels has not been reported in previous literature. The detailed alterations and the variation curve of PSA require further in-depth research in the follow-up to be clearly defined. Postoperative MRI findings demonstrated irregular morphological changes in the prostatic region, consistent with structural alterations following IRE treatment. These results further underscore the therapeutic potential of IRE for managing prostatic hyperplasia. In this study, 2 patients developed postoperative urinary retention, requiring prolonged catheterization. The most severe case resolved with catheter removal 30 days postsurgery. These outcomes highlight the need for careful monitoring and management of urinary retention as a potential postoperative complication of IRE. However, the detailed causes of this adverse reaction and how to avoid it require an expanded sample size and further research to clarify.

IRE surgery demonstrates high efficacy in preserving urinary function and quality of life while significantly reducing intraoperative bleeding compared to conventional treatments for prostatic hyperplasia. While some patients may experience transient declines in sexual function following the procedure, these effects are temporary and typically resolve over time, returning to baseline levels. Additionally, the procedure is associated with an exceptionally low rate of perioperative complications, underscoring its safety profile. However, it is worth noting that the sample size in this study is relatively small. The actual situation may differ somewhat from the results of this study. Further research with a larger sample size is necessary to more accurately determine its safety and effectiveness.

Although IRE offers several advantages, it is not without limitations. One notable challenge is the absence of standardized criteria for the ablation area, which increases the reliance on surgical expertise and may lead to inaccuracies during the procedure, potentially compromising therapeutic efficacy and safety. Additionally, current ablation systems lack real-time feedback, making it difficult to monitor the extent of tissue ablation during surgery. This limitation reduces precision in needle placement and distribution, introducing uncertainty in treatment outcomes. Another limitation is the financial burden associated with IRE, primarily due to the recent adoption of the technology and the cost of disposable electrode needles. The limited development time of IRE ablation has also hindered its widespread application. At present, IRE is predominantly used for the focal treatment of malignant tumors,^[19–22]^ with minimal utilization in benign conditions such as prostatic hyperplasia. This study represents a preliminary investigation into the application and potential of IRE ablation technology for the management of prostatic hyperplasia. However, to establish its safety and efficacy comprehensively, further studies with larger sample sizes and extended follow-up periods are necessary.

In conclusion, this study introduces a novel approach for managing prostate hyperplasia through the application of IRE. The findings suggest that IRE holds significant potential as an effective and well-tolerated therapeutic option, particularly for patients who are unsuitable or unwilling to undergo conventional treatment methods. This innovative technique offers expanded possibilities for clinical practice and patient care. However, the study’s limitations, including the small sample size and short follow-up period, highlight the need for further research. Future studies should focus on larger cohorts and longer follow-up durations to enable a more comprehensive evaluation of IRE’s therapeutic efficacy and functional outcomes. Such efforts will provide a stronger evidence base for integrating IRE into clinical treatment protocols.

## Author contributions

**Data curation:** Bo Chen.

**Investigation:** Yunpeng Guo, Gai Hang.

**Methodology:** Long Chen.

**Project administration:** Quan Wen.

**Validation:** Yuyang Wang.

**Writing – original draft:** Zhiyu Yu.

**Writing – review & editing:** Bo Chen.
